# Cytomegalovirus Viremia, Pneumonitis, and Tocilizumab Therapy

**DOI:** 10.3201/eid1704.101057

**Published:** 2011-04

**Authors:** David van Duin, Cyndee Miranda, Elaine Husni

**Affiliations:** Author affiliation: Cleveland Clinic, Cleveland, Ohio, USA

**Keywords:** Cytomegalovirus, viremia, viruses, biologics, tocilizumab, pneumonitis, rheumatoid arthritis, letter

**To the Editor:** Tocilizumab is a monoclonal antibody that competitively inhibits binding of interleukin-6 (IL-6) to its receptor. It is approved for treatment of rheumatoid arthritis (RA) as monotherapy or with methotrexate. We report a case of cytomegalovirus (CMV) disease complicating treatment with an IL-6 receptor antagonist.

A 41-year-old man who had a diagnosis of nonerosive RA (seronegative for rheumatoid factor and anticyclic citrillinated peptide antibody) in 1994 had fevers in May 2010. Previous treatment included etanercept, methotrexate, and various doses of prednisone (highest dose 40 mg/day). Because of uncontrolled RA, he was treated with monthly infusions of tocilizumab, 600 mg (≈4 mg/kg, first infusion in March 2010 and the second in April 2010), methotrexate (7.5 mg/week), and prednisone (5 mg/day from April 2010 onwards).

Fever, a productive cough with white sputum, and wheezing developed ≈3 weeks after his second infusion of tocilizumab, which resulted in RA symptom resolution (Figure). Tapering of steroid treatment and levofloxacin resulted in some improvement. However, after 1 week, persistent fever led to hospitalization. Worsening shortness of breath, nausea, and vomiting developed. Results of computed tomography (CT) scans of the chest, abdomen, and pelvis were unremarkable. He was transferred to the Cleveland Clinic because of hypotension and intravenous dye–induced renal failure.

**Figure:**
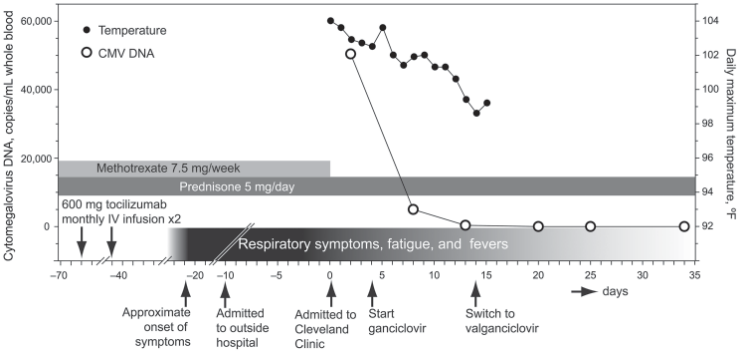
Timeline of events for a 41-year-old man with rheumatoid arthritis. CMV, cytomegalovirus; IV, intravenous.

Daily fever (<103°F), shortness of breath, nausea, and mild diarrhea persisted. After cultures were obtained, he received 1 g vancomycin, 3.375 g piperacillin/tazobactam, and 5 mg/kg lipid amphotericin B (empiric therapy). After a single dose of these drugs, antimicrobial drugs were withheld. Methotrexate and tocilizumab were also withheld. Prednisone (5 mg/day for his duration in the hospital and after discharge) was continued and resulted in resolution of RA symptoms.

Laboratory testing (reference ranges) showed leukocyte count 1,850 cells/μL, hematocrit 26.8%, platelet count 21,000 cells/μL, aspartate aminotransferase 107 U/L (7–40 U/L) alanine aminotransferase 56 U/L (5–50 U/L), alkaline phosphatase 164 U/L (40–150 U/L), and serum creatinine 3.15 mg/dL (0.7–1.4 mg/dL). Testing included negative serologic results for *Bartonella* species, *Coxiella burnetii*, *Histoplasma capsulatum*, *Blastomyces dermatitidis*, *Coccidioides immitis*, hepatitis A, B, and C viruses, HIV, and parvovirus B19; negative PCR results for *H*. *capsulatum* and *Legionella pneumophila* antigens, influenza A/B viruses, respiratory syncytial virus, and human herpesvirus 6; no growth for routine fungal and mycobacterial blood cultures, urine cultures, and stool cultures; negative direct immunofluorescence results for adenovirus, parainfluenza viruses, and human metapneumovirus; and negative stool results for ova and parasites. A bone marrow biopsy did not show any abnormalities.

PCR showed CMV viremia (maximum value 50,413 copies/mL whole blood). A test result for immunoglobulin G against CMV was positive, indicating reactivation of latent infection. Epstein-Barr virus (EBV) viremia was low (1,821 copies/mL whole blood). Although the patient likely showed clinically irrelevant EBV shedding, fatal reactivation of EBV during tocilizumab therapy has been reported (*1*).

Results of CT scans of chest, sinuses, abdomen, and pelvis on admission at our institution were unremarkable. However, scanning of indium 111–labeled leukocytes 12 days after admission showed bilateral pneumonitis, and repeat chest CT showed interval development of ground glass opacities in the right upper lobe.

The patient was treated with intravenous ganciclovir for 10 days at doses adjusted for renal failure. Treatment was changed to oral valganciclovir, 900 mg 2×/d for 20 days, upon discharge. His symptoms gradually improved, and he had no fever after ≈7 days of treatment. His CMV DNA level decreased to 4,996 copies/mL after 3 days of therapy. A negative result for CMV DNA was observed 14 days after starting therapy. Thirty-five days after starting therapy, a CMV DNA test result remained negative, leukocyte count increased to 3,740 cells/μL, hematocrit to 32.3%, and platelet count to 194,000 cells/μL. These findings suggest that pancytopenia was likely secondary to CMV infection. Creatinine level returned to the reference range, and liver enzyme levels improved (aspartate aminotransferase 52 U/L, alanine aminotransferase 73 U/L). Cytopenia and liver toxicity are side effects of treatment with tocilizumab (*2*). His condition showed improvement at follow-up 35 days after starting therapy. He continued to receive prednisone (5 mg/day) and RA symptoms were controlled.

Similar to therapeutic blockade of tumor necrosis factor-α (TNF-α), tocilizumab has been associated with increased risk for infections. Several cases of CMV disease complicating TNF-α blockade, including pneumonia, have been reported (*3*). As in this patient, the effect of steroids on risk for infection often cannot be determined. Given the role of IL-6 in antiviral immunity, CMV reactivation in IL-6 blockade is not surprising (*4*). Frequent adverse events are upper respiratory tract infections, headache, nasopharyngitis, and gastrointestinal symptoms (*4*). Rates of serious infections were 5.3 infections/100 patient-years in placebo-treated patients and 3.9 infections/100 patient-years in patients treated with tocilizumab for 6 months (*2*). This rate was 7.2 infections/100 patient-years after 3 months of TNF-α blockade (*5*). Other opportunistic infections that have been reported in clinical trials include *Pneumocystis jirovecii* pneumonia, herpes zoster, EBV hepatitis, tuberculosis, and asymptomatic *Mycobacterium avium–intracellulare* (*6**–**10*). Thus, CMV disease should be considered when patients receiving tocilizumab have febrile syndromes.
